# Radiographic Evaluation of Medication-Related Osteonecrosis of the Jaw (MRONJ) With Different Primary Cancers and Medication Therapies

**DOI:** 10.7759/cureus.42830

**Published:** 2023-08-01

**Authors:** Kavya Shankar Muttanahally, Aditya Tadinada

**Affiliations:** 1 Department of Adult Restorative Dentistry, Oral and Maxillofacial Radiology, University of Nebraska Medical Center, Lincoln, USA; 2 Department of Oral and Maxillofacial Radiology, University of Connecticut, Farmington, USA

**Keywords:** cone beam computed tomography (cbct), primary cancer, osteonecrosis, mronj, antiresorptive

## Abstract

Background: Medication-related osteonecrosis of the jaw (MRONJ) is a condition that affects the jaws and is characterized by exposed bone in the oral cavity that persists for more than eight weeks despite treatment. Additional criteria include that the patient should have a current or past history of antiresorptive drugs and/or in combination with antiangiogenic drugs, absence of metastasis, and no previous radiotherapy to the affected area. The radiographic features of MRONJ in most instances do not have any specific radiographic features. This is because standard radiographs usually show no stark abnormalities in the early stages of the disease.

Objective: The study aimed to evaluate if any specific radiographic patterns are associated with primary cancers and between medications.

Materials and methods: The study is an observational case series. A total of 50 cases of possible osteonecrosis from June 2010 to June 2013 archives of the Department of Oral and Maxillofacial Radiology were assessed. Based on the history, 12 cases that had a history of medication use that could lead to medication-related osteonecrosis of the jaw (MRONJ) were selected. Cone beam computed tomography (CBCT) scans of these 12 cases were evaluated using the CBCT reconstruction program InVivo Dental version 6 (Anatomage Inc., San Jose, CA, USA). The number of areas showing sequestration, the pattern of osteonecrosis, and the extent were assessed. Primary cancer and the type of medication were also assessed to identify if certain cancers or drugs showed any distinctive pattern of osteonecrosis. Reconstructed panoramic images and true three-dimensional (3D) multi-planar images were assessed to study the condition. An oral and maxillofacial radiology resident in training and a board-certified oral and maxillofacial radiologist assessed the images.

Results: Radiographic findings varied among the 12 cases and included generalized sclerosis, osteosclerosis with widened periodontal ligament (PDL) space, bony sequestra, and bony necrosis. However, no specific or distinctive radiographic patterns were observed in any of the cases, regardless of the type of primary cancer or medication used.

Conclusion: It is challenging to radiographically distinguish between MRONJ cases with different primary cancer and/or medication.

Future directions: Future studies should include evaluating larger samples with varying primary cancers and medications and combination drug therapies. Cases in an advanced stage of MRONJ do not have distinctive features due to extensive destruction and superimposed infection; it may be valuable to evaluate patients in the early stages of MRONJ to better understand distinguishing radiographic patterns specific to certain primary cancers or medications.

## Introduction

Medication-related osteonecrosis of the jaw (MRONJ) was previously referred to as bisphosphonate-related osteonecrosis of the jaw (BRONJ). The American Association of Oral and Maxillofacial Surgeons (AAOMS) defines MRONJ as a debilitating bone disorder of the jaw characterized by persistent exposure of bone in the oral cavity for more than eight weeks refractory to treatment, current or previous history of antiresorptive therapy alone or in combination with immune modulators or antiangiogenics use, no evidence of metastasis, and no prior radiotherapy to the affected region [1,2]. Patients who are experiencing osteoporosis, osteopenia, and multiple forms of cancer are often prescribed agents that inhibit osteoclast resorption of the bone. A calendar year study demonstrated that 5.1 million patients aged 55+ received prescriptions for IV bisphosphonates alone [1]. There is a strong correlation between a history of IV bisphosphonates and denosumab therapies with a risk of medication-related osteonecrosis of the jaw (MRONJ). Studies showed that 70-100/10,000 persons treated with these medications are affected by MRONJ [3,4].

Currently, the stratification of disease presentation is determined through the American Association of Oral and Maxillofacial Surgeons (AAOMS) clinical staging guidelines. The pathophysiology of MRONJ is still not known, but there are several hypotheses to explain the uniqueness of why this condition so frequently occurs in the jaw bones. The proposed hypotheses include altered bone remodeling or over-suppression of bone resorption, angiogenesis inhibition, and constant microtrauma. Immune suppression is also considered a possible reason along with vitamin D deficiency, bisphosphonate toxicity, and inflammation or infection [5-8].

The radiographic features of MRONJ in most instances do not have any specific radiographic features. This is because standard radiographs usually show no stark abnormalities in the early stages of the disease, and a possible decalcification may be present. For this reason, they are poor screening tools for diagnosing this condition [9-11]. While there are no specific radiographic features, localized or diffuse osteosclerosis or thickening of the lamina dura may help in identifying possible sites of exposed necrotic bone [12]. Lack of ossification at a dental extraction site may help as an early radiographic sign. The findings on three-dimensional (3D) imaging such as computed tomography (CT) are also not very specific, but three-dimensional imaging can help in detecting more subtle changes in bone mineralization. Because of its ability to generate cross-sectional evaluation, 3D imaging may be better at identifying areas of focal sclerosis, thickened lamina dura, early sequestrum formation, and any possible reactive periosteal bone.

Limited radiographic parameters exist to aid in the diagnosis of MRONJ, and radiographic findings alone cannot provide adequate data for developing staging. Panoramic imaging (PI) is currently used to screen for osteonecrosis; however, studies have shown this method to be inadequate in screening for MRONJ. Cone beam computed tomography (CBCT) offers the added benefit of 3D imaging for added visualization; however, there is limited evidence to support its use in MRONJ screening [13-15].

The current literature lacks comprehensive studies investigating the potential gap in understanding the specific or distinctive patterns in radiographic presentation between different types of primary cancers, medications, and the radiographic patterns associated with primary cancers. While some individual studies have explored certain aspects of this topic, there is a significant gap in the existing literature when it comes to comprehensive evaluations encompassing multiple primary cancers and their corresponding radiographic patterns. This study aims to evaluate if there are any specific or distinctive patterns in their radiographic presentation between the type of primary cancer, the medications, and the radiographic patterns between primary cancers.

## Materials and methods

**Figure 1:**
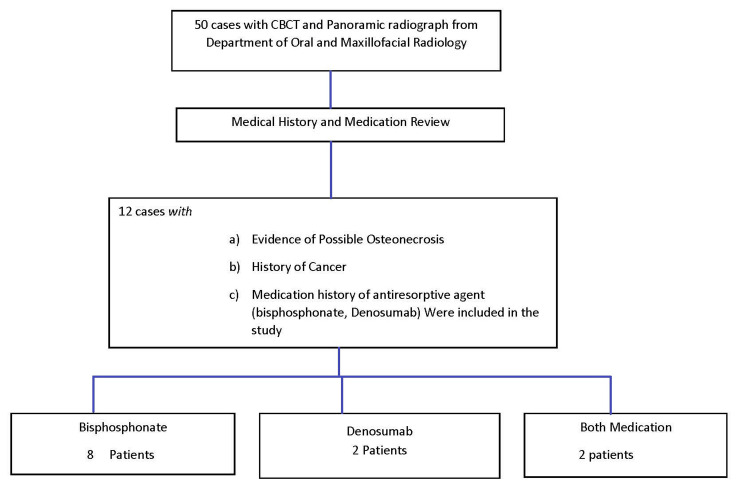
Selection of cases CBCT: cone beam computed tomography

From the archives of the Department of Oral and Maxillofacial Radiology at the University of Connecticut Health Center from June 2010 to June 2013, a total of 50 cases with possible osteonecrosis were evaluated, and based on the history, 12 cases had a history of medication use that could possibly lead to medicine-related osteonecrosis of the jaw (MRONJ). The selection of cases is shown in Figure 1. The inclusion criteria of this study are patients with a history of bisphosphonates and drugs that can cause MRONJ. Patients with no history of drug therapy or medication regimens that could potentially lead to MRONJ were excluded from the study.

In this observational case series, we evaluated if there are any specific or distinctive patterns in their radiographic presentation between the type of primary cancer and between medications. We also evaluated if there were any specific or distinctive patterns of osteonecrosis between medications (Table 1).

**Table 1:** Radiographic presentation between the type of primary cancer and between medications IV: intravenous, PDL: periodontal ligament

Case number	Type of primary cancer	Treatment/medications	Significant radiographic findings
1	Parathyroid cancer	Denosumab	Generalized sclerosis
2	Breast cancer	IV bisphosphonates	Generalized osteosclerosis with widened PDL space
3	Breast cancer	IV bisphosphonates	Generalized osteosclerosis with widened PDL space
4	Lung cancer (stage 4)	IV bisphosphonates	Bony sequestra in the left maxilla
5	Prostate cancer	IV bisphosphonates	Bony sequestra in the maxilla and mandible
6	Bone cancer	IV bisphosphonates	No significant changes
7	Multiple myeloma	IV bisphosphonates	Generalized sclerosis
8	Parathyroid cancer	Denosumab	Bony necrosis in the lower anterior mandible
9	Metastatic breast cancer	IV bisphosphonates	Sequestra in the left mandible
10	Metastatic breast cancer	Denosumab + IV bisphosphonates	Buccal/lingual plates of the anterior mandible
11	Multiple myeloma	IV bisphosphonates	Severe osteosclerotic changes in the anterior maxilla
12	Prostate cancer with bone metastasis	Denosumab + IV bisphosphonates	Unhealed sockets with no sclerotic changes

MiPACS Dental Enterprise Viewer 3.2.2 (Medicor Imaging, Charlotte, NC, USA) and InVivo Dental version 6 (Anatomage Inc., San Jose, CA, USA) programs were used to evaluate the 2D and 3D images, respectively. The number of areas showing osteonecrosis, varying patterns of osteonecrosis if present, and the extent were evaluated. Primary cancer and the type of medication were also evaluated to identify if certain cancers or drugs showed any distinctive osteonecrosis pattern. Reconstructed panoramic images were compared to true three-dimensional multi-planar imaging to establish any additional contribution to evaluating the condition. An oral and maxillofacial radiology resident in training and a board-certified oral and maxillofacial radiologist evaluated the images.

Statistical analysis

In this study, descriptive statistical analysis was performed to comprehensively understand the collected data. Descriptive statistics were used to summarize and describe the key characteristics, trends, and patterns observed in the dataset.

## Results

CBCT scans were evaluated in the axial, sagittal, and coronal planes. Some of the key features evaluated were patterns of osteonecrosis (regions or presentation), pathological fractures, and bone sequestra. The evaluation of the presented cases demonstrated that multi-planar imaging was significantly superior to reconstructed panoramic imaging in assessing the extent of lesions and localizing sequestra.

The descriptive statistics for the collected cases are as follows. There are 12 cases with a history of medication use that could lead to medication-related osteonecrosis of the jaw. The primary cancers were as follows: parathyroid cancer (two cases), breast cancer (three cases), lung cancer (stage 4) (one case), prostate cancer (two cases), bone cancer (one case), multiple myeloma (two cases), metastatic breast cancer (two cases), and prostate cancer with bone metastasis (one case). The treatments used were denosumab (four cases) and IV bisphosphonates (eight cases). The significant radiographic findings were as follows: generalized sclerosis (three cases), generalized osteosclerosis with widened periodontal ligament (PDL) space (two cases), bony sequestra in the left maxilla (one case), bony sequestra in the maxilla and mandible (one case), bony necrosis in the lower anterior mandible (one case), sequestra in the left mandible (one case), buccal/lingual plates of the anterior mandible (one case), severe osteosclerotic changes in the anterior maxilla (one case), unhealed sockets #3, #8, #9 and no sclerotic changes (one case), and no significant changes (one case).

**Figure 2:**
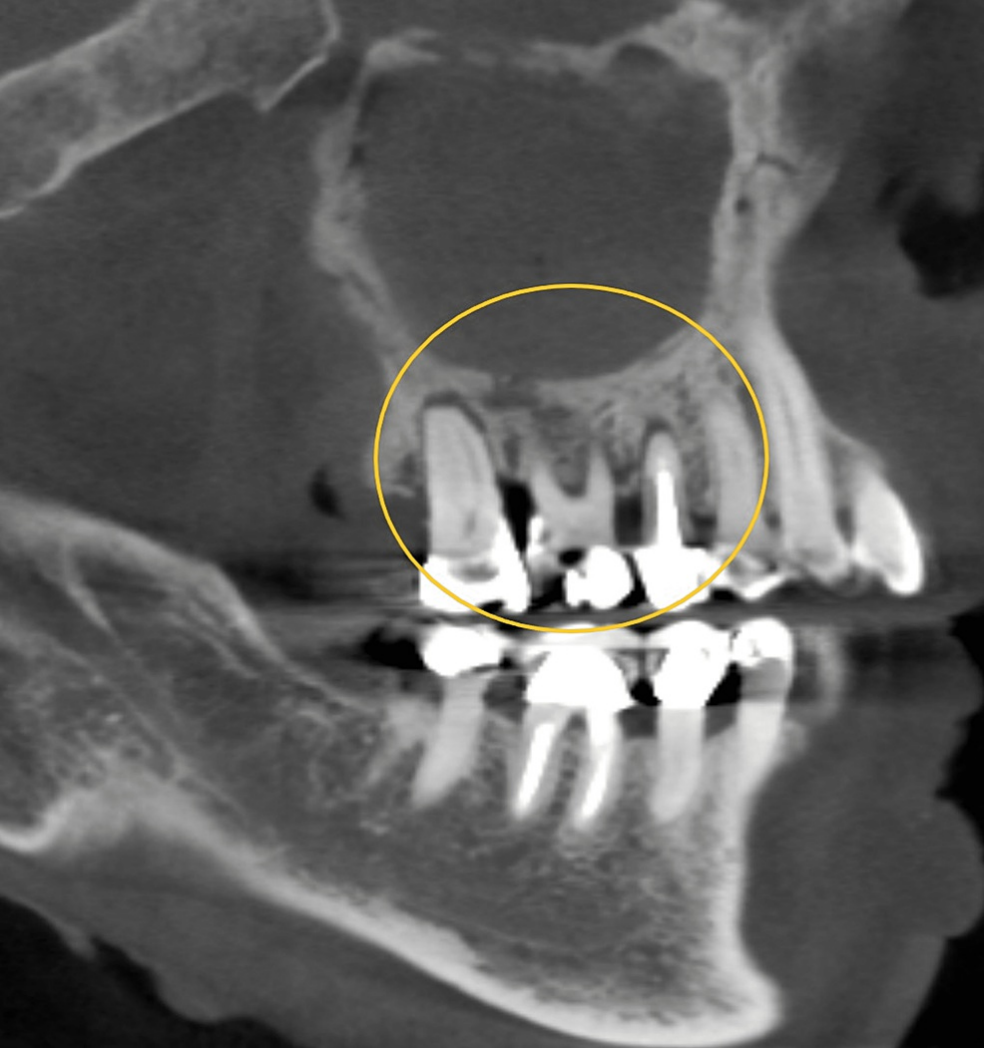
Generalized osteosclerosis with widened PDL space (yellow circle) PDL: periodontal ligament

**Figure 3:**
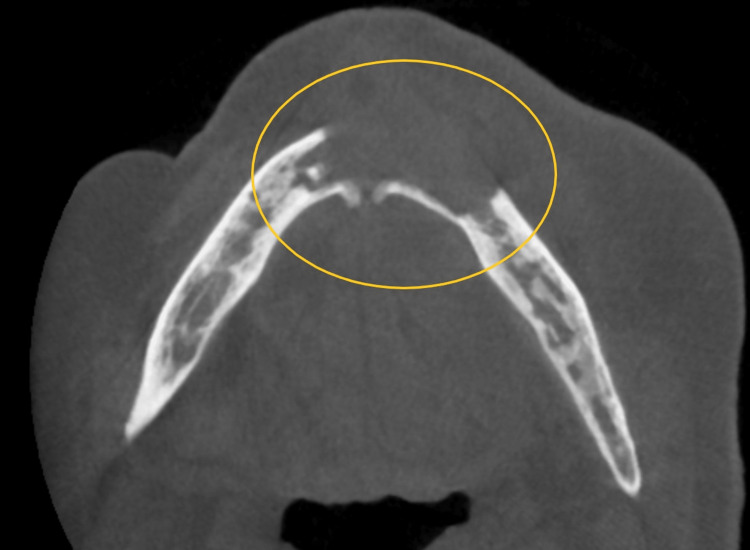
Bony sequestra in the mandible along with discontinuity in the buccal cortical plate (yellow circle)

**Figure 4:**
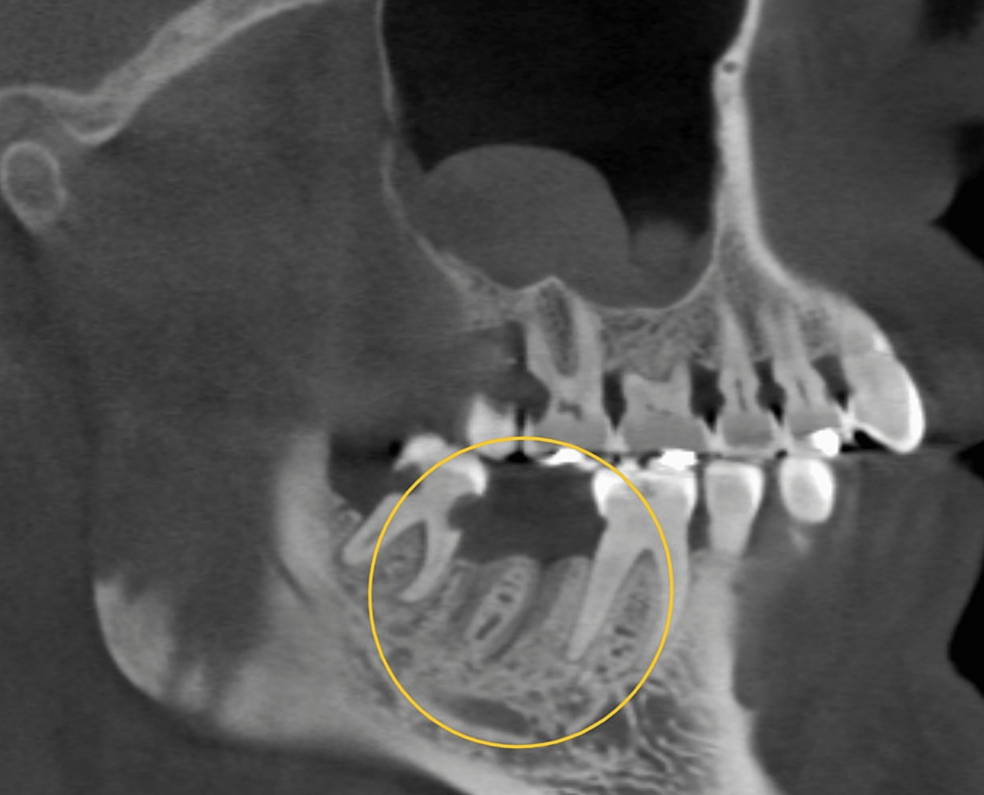
Unhealed sockets without sclerotic changes (yellow circle)

In this set of cases, parathyroid cancer was treated with denosumab, a medication that resulted in generalized sclerosis on radiographs. Breast cancer cases were treated with IV bisphosphonates and showed significant radiographic findings of generalized osteosclerosis with widened periodontal ligament (PDL) space (Figure 2). Two breast cancer cases also presented bony sequestrum. A patient with stage 4 lung cancer had bony sequestra in the left maxilla, while a prostate cancer patient exhibited bony sequestra in the maxilla and mandible. Another patient with bone cancer did not show any significant radiographic changes. Multiple myeloma cases displayed either generalized sclerosis or severe osteosclerotic changes in the anterior maxilla. A case of parathyroid cancer treated with denosumab showed bony necrosis in the lower anterior mandible. Metastatic breast cancer cases presented with sequestra in the mandible (Figure 3), and one case also showed discontinuity in the buccal and lingual plates. A prostate cancer patient with bone metastasis exhibited unhealed sockets without sclerotic changes (Figure 4). Radiographic findings varied among the cases and included generalized sclerosis, osteosclerosis with widened PDL space, bony sequestra, and bony necrosis. However, no specific or distinctive radiographic patterns were observed in any of the cases, regardless of the type of primary cancer or medication used.

## Discussion

MRONJ, a significant oral complication, was first reported in 2003. The American Association of Oral and Maxillofacial Surgeons has provided specific criteria for the diagnosis of MRONJ as well as a staging system. The staging and criteria are primarily based on clinical evaluation with stage 0 being based on radiographic examination (AAOMS staging) [1]. While the primary presentation and diagnosis including the staging may be based on clinical evaluation, there is significant value to radiographic examination, especially for treatment planning [16-18].

Osteonecrosis is characterized by sequestrum formation, and if the systemic health is good and if there is adequate time, the body launches its defense mechanism leading to periosteal new bone formation primarily aimed at preventing a pathological fracture. The uniqueness of this feature is that the new bone is on the outside or on the periosteal side. Typically, the new bone is in the innermost region of the bone. Bacterial and radiation-induced necrosis of the jaw can be discerned from each other based on specific identifying features. In bacterial osteonecrosis, along with sequestrum formation, periosteal new bone formation is an expected radiographic finding. This is when compared to osteoradionecrosis. Because of the annihilation of blood supply to the area where therapeutic radiation has been performed, there is typically no new bone formation. This is a very valuable diagnostic feature [19,20].

With regard to chemically induced osteonecrosis such as medication-related osteonecrosis, there are several drugs in varying categories that are used for the treatment of various tumors such as prostate cancer or breast cancer, or for different systemic conditions such as osteoporosis and Paget’s disease [21,22]. A key question is whether there is a specific pattern of osteonecrosis that is visualized upon radiographic imaging that is specific to either the primary tumor or the specific medication. This can help in the identification of both the primary tumor and the medication being used.

There are not an adequate number of well-designed studies that have evaluated if the primary tumor or the condition for which medication is being taken has any specific radiographic pattern that can be discernible from the radiographic presentation of other tumors or conditions and if these can be specifically associated to these conditions or to the specific medication [23,24]. In our current study, evaluation of the medical history, medication history, and radiographic analysis demonstrated that there was insufficient evidence to distinguish radiographic patterns among patients with similar primary cancer. However, when the results were stratified by medication class, it appears that intravenous bisphosphonate therapy demonstrated more severe osteonecrosis on imaging. The strength of the association between intravenous bisphosphonate use and osteonecrosis of the jaw (ONJ) has been evaluated in various studies, primarily in cancer patients receiving high-dose intravenous bisphosphonates. The findings have shown an increased risk of ONJ in this specific population, although the absolute risk remains relatively low. It is important to note that osteonecrosis occurring in other bones, such as long bones or the hip, is less commonly associated with bisphosphonate use. However, some studies have suggested a potential association, particularly with long-term or high-dose use of intravenous bisphosphonates [22]. However, there were no specific or distinctive radiographic patterns observed in the presentation of the area of MRONJ between different types of primary cancer or medication.

The study had limitations in sample size and limited clinical information. Future research should follow patients longitudinally, starting in the early stages of MRONJ (stages 0-1). With larger sample sizes as part of possibly multicenter studies, this is a useful direction for future studies because the number of well-documented cases and the incidence rates of MRONJ are low.

Despite over a decade of knowledge on MRONJ, there are still reports in the literature describing the clinical symptoms of MRONJ without exposed necrotic bone, known as stage 0 disease, which is considered to be at risk for MRONJ [24]. Case series reports have been published, characterizing radiographic findings in patients with symptomatic stage 0, contributing to efforts to identify radiographic patterns that may precede the development of MRONJ in at-risk populations.

## Conclusions

There are no specific patterns between primary tumors or conditions and medications associated with the radiographic appearance of MRONJ. It is challenging to radiographically distinguish MRONJ between primary cancers, but IV bisphosphonates demonstrated more severe osteonecrosis. Future directions should include evaluating larger samples with varying primary cancers and medications. Advanced MRONJ cases do not have distinctive features due to extensive destruction and superimposed infection; it may be valuable to evaluate cases in the early stages of MRONJ to better understand distinguishing radiographic patterns specific to certain primary cancers or medications.

## References

[1] Ruggiero SL, Dodson TB, Aghaloo T, Carlson ER, Ward BB, Kademani D (2022). American Association of Oral and Maxillofacial Surgeons’ position paper on medication-related osteonecrosis of the jaws-2022 update. J Oral Maxillofac Surg.

[2] Wan JT, Sheeley DM, Somerman MJ, Lee JS (2020). Mitigating osteonecrosis of the jaw (ONJ) through preventive dental care and understanding of risk factors. Bone Res.

[3] Feng X, McDonald JM (2011). Disorders of bone remodeling. Annu Rev Pathol.

[4] Uyanne J, Calhoun CC, Le AD (2014). Antiresorptive drug-related osteonecrosis of the jaw. Dent Clin North Am.

[5] Al-Eid R, Alduwayan T, Bin Khuthaylah M, Al Shemali M (2020). Dentists' knowledge about medication-related osteonecrosis of the jaw and its management. Heliyon.

[6] AlDhalaan NA, BaQais A, Al-Omar A (2020). Medication-related osteonecrosis of the jaw: a review. Cureus.

[7] Lorenzo-Pouso AI, Bagán J, Bagán L (2021). Medication-related osteonecrosis of the jaw: a critical narrative review. J Clin Med.

[8] Calvani F, Cutone A, Lepanto MS, Rosa L, Valentini V, Valenti P (2018). Efficacy of bovine lactoferrin in the post-surgical treatment of patients suffering from bisphosphonate-related osteonecrosis of the jaws: an open-label study. Biometals.

[9] Drake MT, Clarke BL, Khosla S (2008). Bisphosphonates: mechanism of action and role in clinical practice. Mayo Clin Proc.

[10] Gralow JR, Biermann JS, Farooki A (2013). NCCN task force report: bone health in cancer care. J Natl Compr Canc Netw.

[11] Cardoso CL, Barros CA, Curra C (2017). Radiographic findings in patients with medication-related osteonecrosis of the jaw. Int J Dent.

[12] Ruggiero SL, Dodson TB, Assael LA, Landesberg R, Marx RE, Mehrotra B (2009). American Association of Oral and Maxillofacial Surgeons position paper on bisphosphonate-related osteonecrosis of the jaws--2009 update. J Oral Maxillofac Surg.

[13] Williams WB, O'Ryan F (2015). Management of medication-related osteonecrosis of the jaw. Oral Maxillofac Surg Clin North Am.

[14] Aghaloo T, Hazboun R, Tetradis S (2015). Pathophysiology of osteonecrosis of the jaws. Oral Maxillofac Surg Clin North Am.

[15] Stockmann P, Hinkmann FM, Lell MM, Fenner M, Vairaktaris E, Neukam FW, Nkenke E (2010). Panoramic radiograph, computed tomography or magnetic resonance imaging. Which imaging technique should be preferred in bisphosphonate-associated osteonecrosis of the jaw? A prospective clinical study. Clin Oral Investig.

[16] Obinata K, Shirai S, Ito H (2017). Image findings of bisphosphonate related osteonecrosis of jaws comparing with osteoradionecrosis. Dentomaxillofac Radiol.

[17] Chiandussi S, Biasotto M, Dore F, Cavalli F, Cova MA, Di Lenarda R (2006). Clinical and diagnostic imaging of bisphosphonate-associated osteonecrosis of the jaws. Dentomaxillofac Radiol.

[18] Hutchinson M, O'Ryan F, Chavez V (2010). Radiographic findings in bisphosphonate-treated patients with stage 0 disease in the absence of bone exposure. J Oral Maxillofac Surg.

[19] Mallya SM, Tetradis S (2018). Imaging of radiation- and medication-related osteonecrosis. Radiol Clin North Am.

[20] Rocha GC, Jaguar GC, Moreira CR, Neves EG, Fonseca FP, Pedreira EN (2012). Radiographic evaluation of maxillofacial region in oncology patients treated with bisphosphonates. Oral Surg Oral Med Oral Pathol Oral Radiol.

[21] Beth-Tasdogan NH, Mayer B, Hussein H, Zolk O (2017). Interventions for managing medication-related osteonecrosis of the jaw. Cochrane Database Syst Rev.

[22] Kühl S, Walter C, Acham S, Pfeffer R, Lambrecht JT (2012). Bisphosphonate-related osteonecrosis of the jaws--a review. Oral Oncol.

[23] Junquera L, Gallego L (2008). Nonexposed bisphosphonate-related osteonecrosis of the jaws: another clinical variant?. J Oral Maxillofac Surg.

[24] Patel S, Choyee S, Uyanne J (2012). Non-exposed bisphosphonate-related osteonecrosis of the jaw: a critical assessment of current definition, staging, and treatment guidelines. Oral Dis.

